# Development and validation of an individualized immune prognostic model in stage I–III lung squamous cell carcinoma

**DOI:** 10.1038/s41598-021-92115-0

**Published:** 2021-06-16

**Authors:** Qi-Fan Yang, Di Wu, Jian Wang, Li Ba, Chen Tian, Yu-Ting Liu, Yue Hu, Li Liu

**Affiliations:** 1 Cancer Center, Union Hospital, Tongji Medical College, Huazhong University of Science and Technology, Wuhan, 430022 China; 2 Department of Ultrasound, Union Hospital, Tongji Medical College, Huazhong University of Science and Technology, Wuhan, 430022 China

**Keywords:** Tumour biomarkers, Cancer microenvironment, Cancer

## Abstract

Lung squamous cell carcinoma (LUSC) possesses a poor prognosis even for stages I–III resected patients. Reliable prognostic biomarkers that can stratify and predict clinical outcomes for stage I–III resected LUSC patients are urgently needed. Based on gene expression of LUSC tissue samples from five public datasets, consisting of 687 cases, we developed an immune-related prognostic model (IPM) according to immune genes from ImmPort database. Then, we comprehensively analyzed the immune microenvironment and mutation burden that are significantly associated with this model. According to the IPM, patients were stratified into high- and low-risk groups with markedly distinct survival benefits. We found that patients with high immune risk possessed a higher proportion of immunosuppressive cells such as macrophages M0, and presented higher expression of CD47, CD73, SIRPA, and TIM-3. Moreover, When further stratified based on the tumor mutation burden (TMB) and risk score, patients with high TMB and low immune risk had a remarkable prolonged overall survival compared to patients with low TMB and high immune risk. Finally, a nomogram combing the IPM with clinical factors was established to provide a more precise evaluation of prognosis. The proposed immune relevant model is a promising biomarker for predicting overall survival in stage I–III LUSC. Thus, it may shed light on identifying patient subset at high risk of adverse prognosis from an immunological perspective.

## Introduction

Lung cancer remains the leading cause of cancer-related death worldwide, with an estimated 19% 5-year overall survival rate in the United States^1^. Lung squamous cell carcinoma (LUSC) is a highly aggressive subtype of non-small cell lung cancer (NSCLC), accounting for approximately 30% of all cases^2^. For patients with resectable LUSC, including stage I–III, relapse is the most common cause of failure^3^. Biomarkers based on gene expression signature can reliably estimate patient survival and represent a potentially significant adjunct^4^. Therefore, identification of patients with high risk for recurrence and death is urgently needed.

Accumulating evidence suggests that molecular feature of the tumor microenvironment (TME) has developed as a promising candidate during cancer formation and progression^5,6^. Immune evasion has been considered as an emerging hallmark of cancer^7,8^. Recently, immunotherapy targeting immune checkpoints has made remarkable strides in improving LUSC patient survival^9–11^. Certain predictive biomarkers, such as tumor-infiltrating lymphocytes, mutational or neoantigen burden, and T-cell receptor repertoire, are also correlated with prognosis^12–17^. The prevailing view highlights the concept that LUSC exhibits different compositions and functions of the TME^18,19^. Thus, the molecular signature characterizing immune infiltration in the TME remains to be further investigated^5,20–22^.

Here, we integrated multiple datasets with gene expression to construct and validate an individualized immune-related prognostic model (IPM) for stage I–III LUSC based on immunological genes. Then, we described immune infiltration and mutation burden that are significantly associated with this signature. Further, a more comprehensive investigation was conducted to enhance the predictive value for LUSC prognosis by combing the IPM with clinical characteristics.

## Methods

### Data acquisition and study population

The gene expression profile and corresponding clinical data of LUSC were retrospectively collected from five public NSCLC cohorts, including one RNA-Seq dataset from The Cancer Genome Atlas (TCGA) LUSC cohort and four microarray datasets from Gene Expression Omnibus (GEO). Only patients with stage I–III squamous carcinoma and complete clinical data were included. We excluded patients with less than ninety-days follow-up. The overview of study design is shown in Fig. S1 and the study characteristics of the included datasets are described in Table S1.

### Gene expression data preprocessing

The TCGA LUSC dataset was selected for the training set, which included 440 LUSC and 49 normal tissue samples. For GEO cohorts, the datasets derived from the same microarray platform were combined as validation datasets to improve the statistical power and then eliminated batch effects using the ComBat function by the sva R package (version: 3.28.0)^23^. Gene expression values were log-transformed and Z-score standardized before removing batch effects for comparable within the same platform.

### Development and validation of the IPM

We developed a prognostic signature from immune-related genes (IRGs) derived from the ImmPort database^24^. First, identification of differentially expressed IRGs between LUSC and normal tissue samples was performed using the limma R package (version: 3.36.5)^25^ with thresholds of false discovery rate (FDR) < 0.05 and log2 |fold change|> 1. Next, we employed univariate Cox analysis to screen IRGs associated with survival using the survival R package (version: 3.1–8)^26^. Finally, we established the IPM according to the expression value multiplied by the regression coefficient, which was determined by a stepwise variable-selecting procedure in the multivariable Cox regression model. The risk score formula was as follows: $Risk\;score = ~\mathop \sum \limits_{{i = 1}}^{n} \beta iXi$, where β*i* stands for the coefficient of individual gene and $Xi$ represents gene expression value (Z-score). Then we divided patients into high- or low-risk groups based on the median risk score derived from the training set. Accordingly, the IPM was further calculated in the two validation datasets, respectively.

### Estimation of the immune infiltration

To quantify the relative proportion of 22 infiltrating immune cell subtypes in complex tumor tissue, we utilized the CIBERSORT algorithm running with the LM22 signature matrix^27^. The sum of the fractions for all estimated cell types was set equal to 1 in each tumor sample. Spearman correlations between risk score and the proportion of infiltrating immune cell subtypes were calculated and visualized using the ggstatsplot R package (version: 0.1.4)^28^. The expression levels were normalized by log_2_(TPM + 1), where TPM denotes transcripts per million. The Human Protein Atlas database was used to verify the protein function by immunohistochemistry.

### Function and pathway enrichment analysis

To obtain the biological understanding of our prognostic model, enrichment analysis of the component IRGs was performed with DAVID (version 6.8)^29^. We visualized the significant biological processes and pathways using the GOplot (Version: 1.0.2)^30^ and ggalluvial (Version: 0.11.1)^31^ R package, respectively.

### Immunohistochemical scores

Immunohistochemistry staining was semi-quantitatively evaluated based on the percentage of positive cells and stain intensity. The percentage of positive cells was categorized as 0 (< 5%), 1 (5–25%), 2 (26–50%), 3 (51–75%), 4 (76–100%). Stain intensity was scored as 0 (negative), 1 (weak), 2 (moderate), 3 (strong). The immunohistochemical staining score was calculated by multiplying the two scores.

### Mutation analysis

In this study, tumor mutation burden (TMB) was defined as the count of nonsynonymous coding mutations per megabase (Mb). Mutation annotation format was downloaded from the GDC data portal and visualized using the maftools R package (version: 1.8.10)^32^. Patients were stratified into high TMB (> 4 mutations/Mb) and low TMB (≤ 4 mutations/Mb) groups based on the previous research to define the TMB threshold for personalized immunotherapy in NSCLC^33^. To determine how the immune-related pathways differ between the two groups, gene set enrichment analysis (GSEA) was performed using Java GSEA software (version 4.0.3)^34^. The normalized enrichment score (NES) and nominal p value were primary statistics for evaluating the enrichment results.

### Construction and evaluation of a predictive nomogram

We combined IPM with clinical variables (including TMB, gender, age, stage) in univariate and multivariate Cox regression analyses. To assess the survival probability of 1-, 3-, 5- year for patients with LUSC, a nomogram was formulated with the rms R package (version: 5.1–4)^35^ on the basis of multivariate analysis results. In addition, the concordance index (C-index) and ROC curve were utilized to evaluate the prediction accuracy of the nomogram and individual prognostic factors. The calibration curve was performed by comparing the prediction of nomogram with the actual observation after bias correction.

### Statistical analysis

Kaplan–Meier survival analysis was performed by survminer R package (version: 0.4.6)^36^, and differences in overall survival between groups were compared using the log-rank method. The receiver operating characteristic (ROC) curve was performed to verify the predictive ability of the IPM by timeROC R package (version: 0.3)^37^. All statistical analyses were performed using R (V3.5.1), GraphPad Prism 7.0, or SPSS 24.0. All statistical significance level was accepted at a two-sided p value < 0.05 in this study.

## Results

### Construction of the IPM

**Figure 1:**
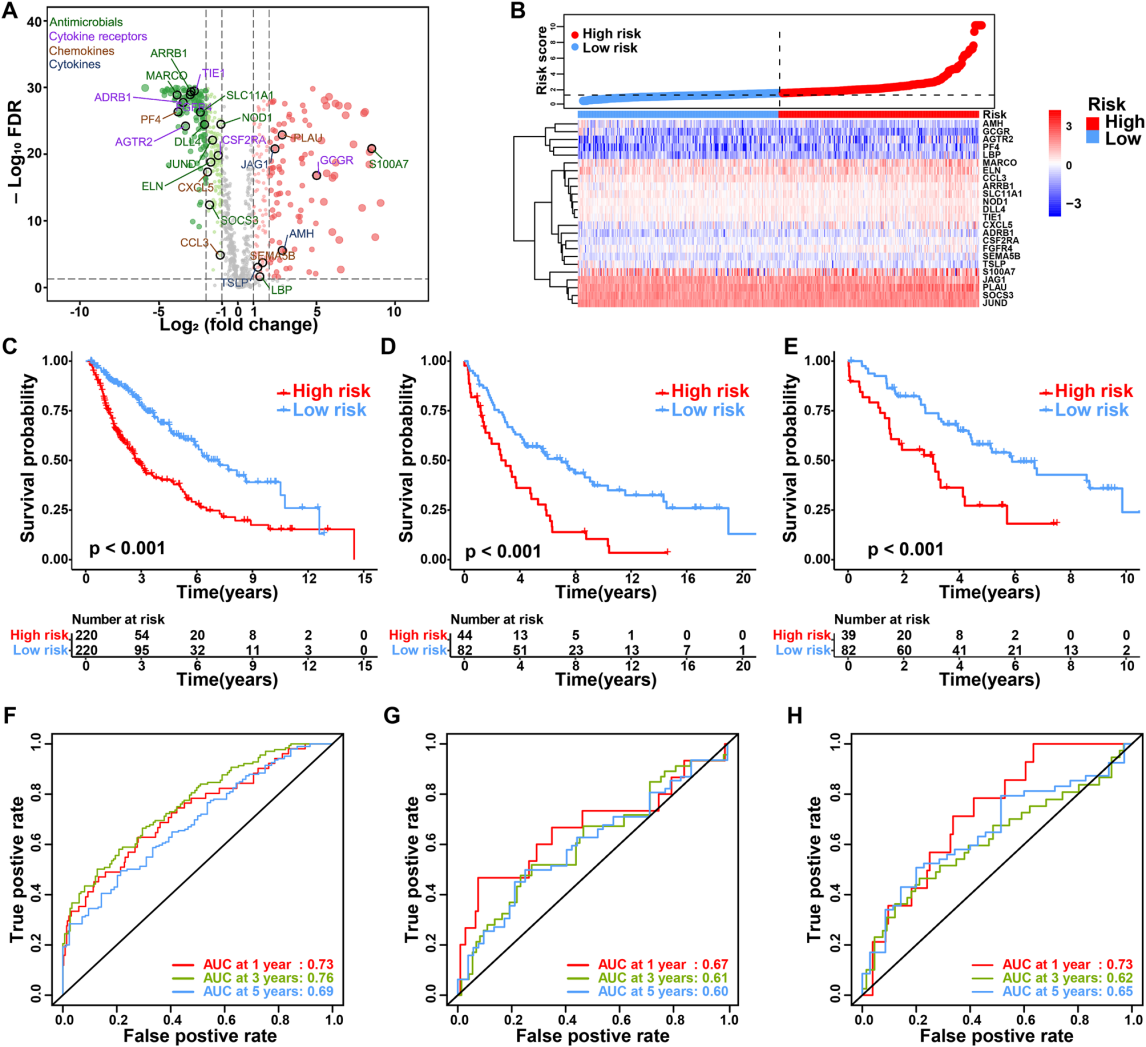
Prognostic analysis of the IPM. (**A**) Volcano plot of differentially expressed IRGs between tumor and normal tissues. Twenty-four candidates selected for the construction of the IPM are indicated. Gene categories associated with the immune process are shown in different colors. (**B**) Distribution of risk score and gene expression normalized by Z-score. Kaplan–Meier plots and time-dependent ROC curves according to the IPM in the training set (**C**,**F**), validation set 1 (**D**,**G**) and validation set 2 (**E**,**F**). (**C**–**E**) Patients in the high-risk group suffered shorter overall survival (p < 0.001, log-rank test). (**F**–**H**) Time-dependent ROC curve validation of the prognostic value of the IPM.

A total of 687 patients with stage I–III LUSC (502 men [73.1%], 185 women [26.9%]; mean age ± SD, 66.7 ± 8.5) were included in this study. For the training set, 465 IRGs were differentially expressed between LUSC and normal tissue samples among 1792 IRGs from the ImmPort database (Fig. 1A). Sixty-one IRGs significantly associated with overall survival were identified after the univariable Cox analysis (Table S2). We developed the IPM according to the combined effect of twenty-four IRGs extracted from the stepwise multivariate Cox regression model (Fig. S2) (Table S3). On the basis of the median immune risk score in the training set, patients were stratified into high- and low-risk groups. The gene expression information and risk distribution are shown in Fig. 1B.

### Validation of the IPM

As shown in Fig. 1C, patients with high risk score indicated a significantly worse prognosis than those with low risk score. We performed time-dependent ROC curves to assess the prognostic accuracy of the IPM. The area under curve (AUC) of the prognostic model for overall survival was 0.73 at 1-year, 0.76 at 3-years, 0.69 at 5-years, respectively, in the training set (Fig. 1F). To validate the prognostic power of the IPM, its performance was further evaluated in two independent validation sets. Applying the same algorithm and cutoff value, the IPM divided patients into two groups with high- and low-risk. Similarly, worse prognoses were also shown to correlate with higher risk scores for patients in two validation sets (Fig. 1D,E). The AUC was 0.67, 0.61, 0.60 and 0.73, 0.62, 0.65 in two validation sets, respectively, which showed similar results in predictive ability (Fig. 1G,H). To eliminate the effects of subsequent treatment, we further explored the prognostic performance of the IPM in progression-free survival for LUSC patients. Consistently, patients with high risk displayed worse progression-free survival (Fig. S3).

### Immune landscape between the high- and low-risk LUSC patients

**Figure 2:**
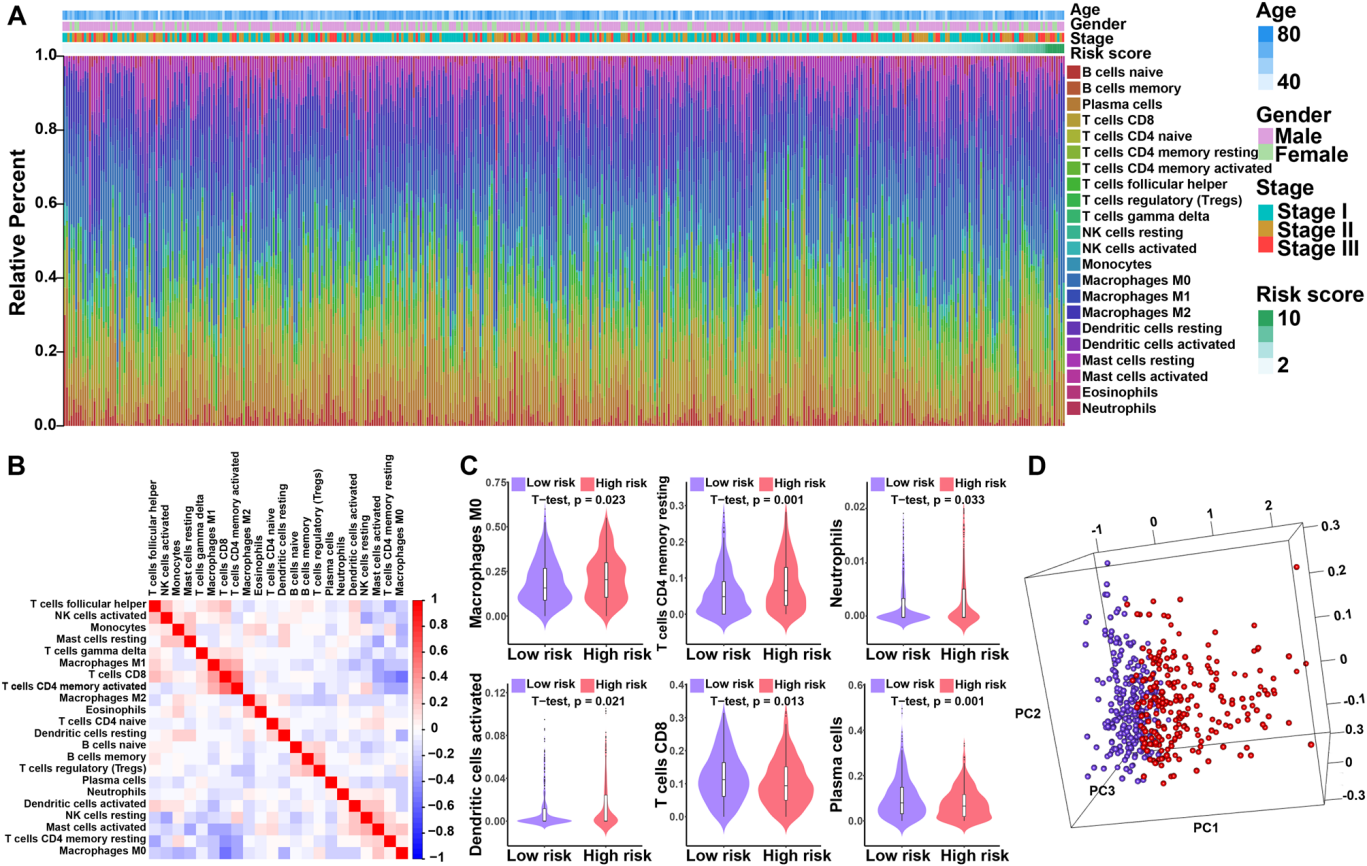
Characterization of immune infiltration in high- and low-risk LUSC patients. (**A**) The relative proportion of 22 infiltrated immune cells in LUSC. (**B**) Correlation analysis among each immune cell proportions. (**C**) Violin plots of distribution of significantly different cell subtypes. Significances were measured using the unpaired t-test. (**D**) Principal components analysis based on the above significantly different immune cells between high- and low-risk LUSC patients.

Using CIBERSORT algorithm, we evaluated the differences in infiltrating immune cells between high- and low-risk LUSC patients. Figure 2A summarizes the results obtained from 440 LUSC patients. Apparently, the proportion of immune cells in LUSC varies between and within groups (Fig. 2A). Thus, variations in immune cell proportions probably depicted the intrinsic feature which identifies individual differences. Besides, the percentages of different immune cell subpopulations were moderately correlated (Fig. 2B).

The high-risk LUSC patients had significantly higher proportions of macrophages M0, dendritic cells activated, T cells CD4 memory resting, neutrophils, and dendritic cells activated, whereas T cells CD8 and plasma cells proportions were relatively lower (Fig. 2C). Similar results were observed for the correlation between risk score and immune cells (Fig. S4). Indeed, the samples of high- and low-risk patients were distinct group‐bias clustering by principal components analysis based on the above significantly different cell subpopulations (Fig. 2D). Taken together, these results surmised that the heterogeneity of immune infiltration in LUSC may serve as a decisive factor that regulates the progress of immunotherapy of cancer.

**Figure 3:**
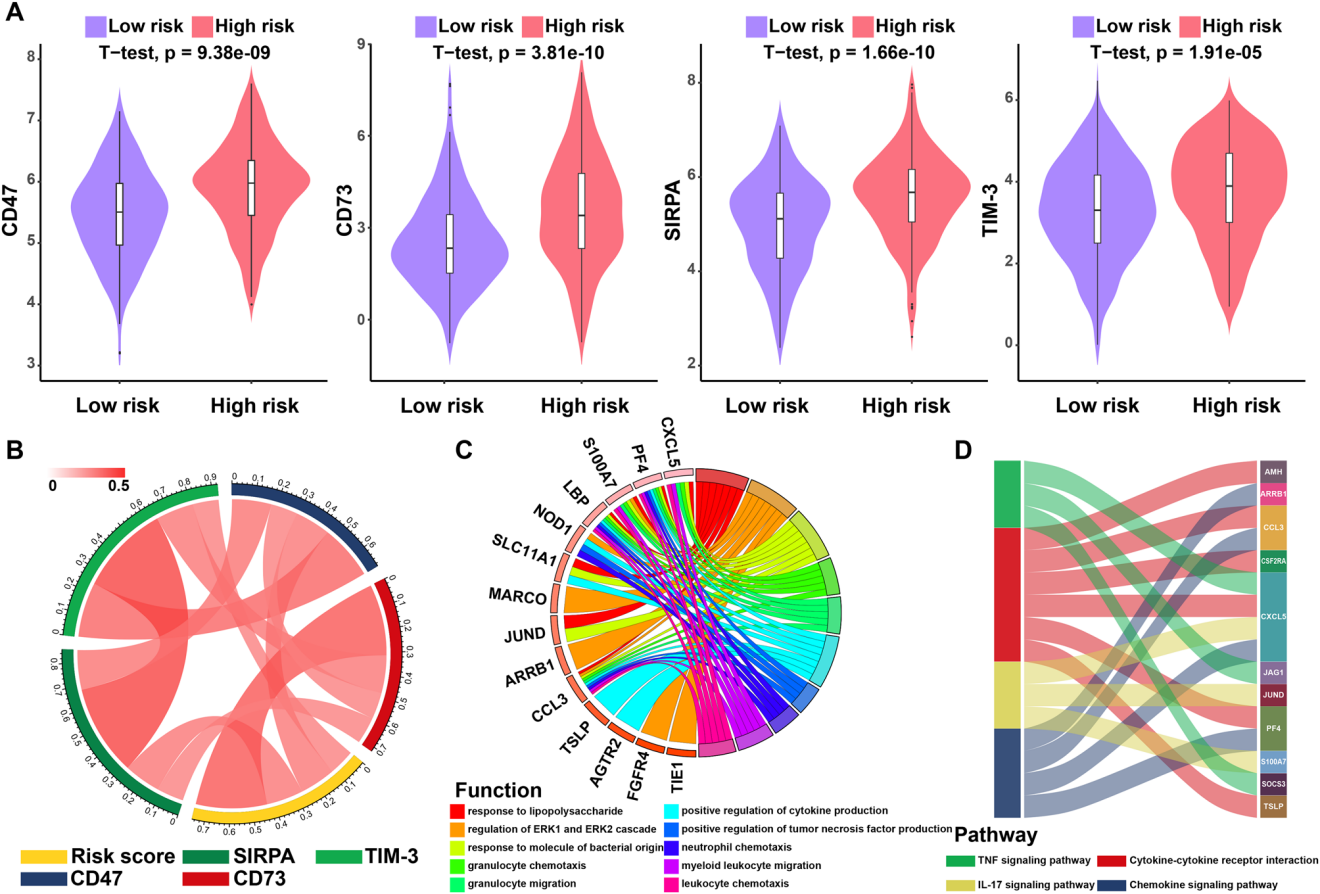
Function and pathway enrichment of the immune prognostic model. (**A**) Violin plots of the expression of immune checkpoints between high- and low-risk groups. Significances were measured using the unpaired t-test. (**B**) Correlation of the expression of immune checkpoints with risk scores. (**C**) Circular visualization of the GO function enrichment of biological processes for the IRGs. (**D**) Sankey plot of the KEGG pathway enrichment for the IRGs.

Tumor-associated macrophage (TAM) therapy aimed at novel TAM targets for modulation of TME showed promising preclinical results^38^. We observed the relationship between risk score and emerging biomarkers for immune checkpoints. Our results showed that the expression of critical TAM targets (CD47, CD73, SIRPA, and TIM-3), which positively correlated with immune risk score, were significantly upregulated in the high-risk group, portending that the worse prognosis of high-risk LUSC patients is partly because of the immunosuppressive microenvironment (Fig. 3A,B) (Table S4). Additionally, immunohistochemistry staining results validated from the Human Protein Atlas database revealed the CD47, CD73, SIRPA, and TIM-3 protein to be upregulated in lung cancer tissues compared to normal lung tissues (Fig. S5).

### Function and pathway enrichment of the IPM

To further investigate the underlying biological effects of the IPM, we performed GO and KEGG enrichment analyses to reveal the function and pathway of the twenty-four survival-related IRGs (Fig. 3C,D) (Table S4). Based on these results, the IRGs were mainly enriched in immune response, migration of immune cells, and immune signaling pathway (Fig. 3C,D) (Table S5). These results depicted the hyperactive immune reaction in the LUSC patients with high immune risk.

### Relationship between mutational signature and the IPM

**Figure 4:**
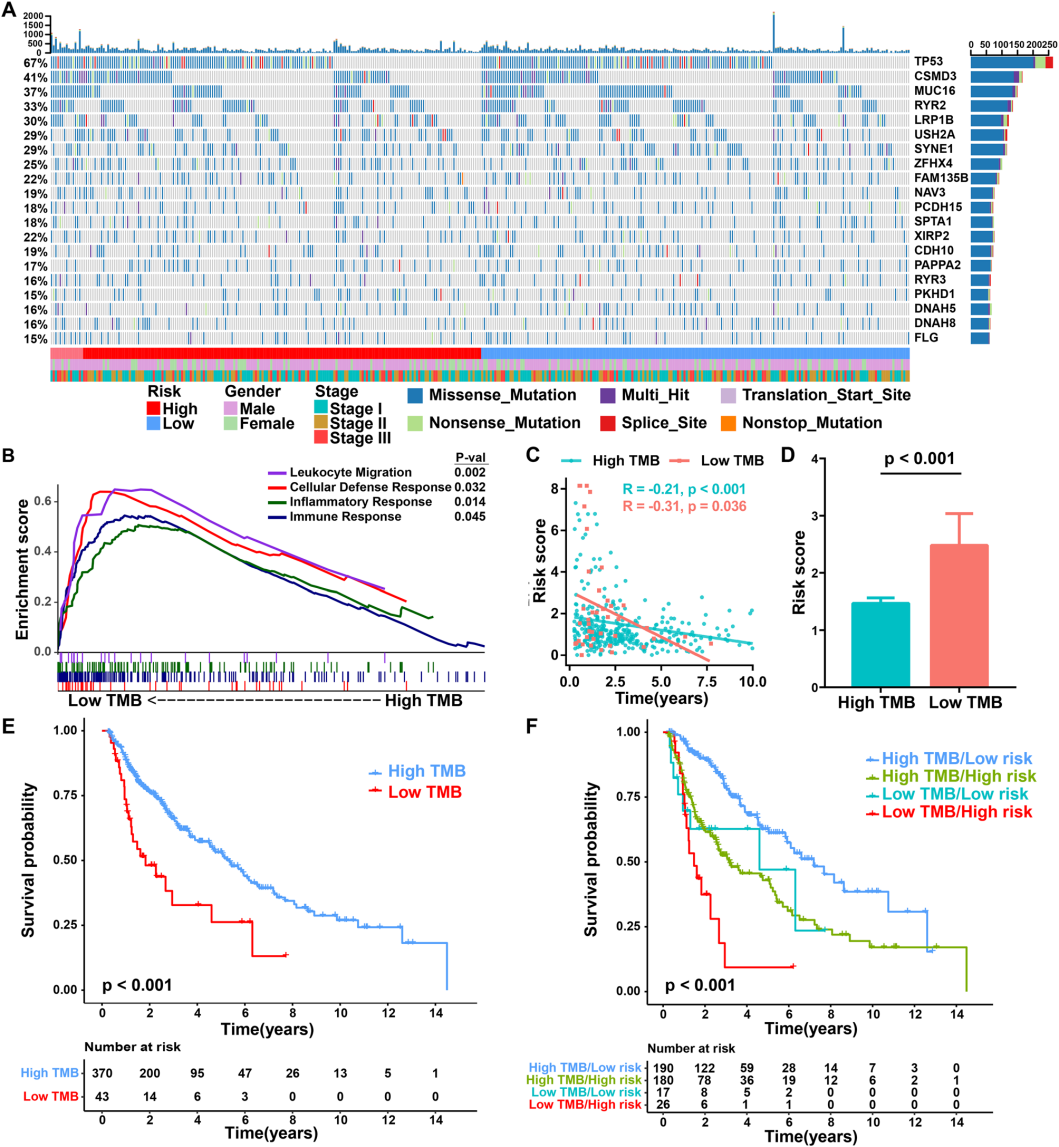
Relationship between mutational signature and the IPM. (**A**) Stacked plots of mutational frequency in individual tumors (histogram, top), mutations in the top 20 genes (tile plot, middle), their mutational counts (histogram, right) and mutational types (bottom). (**B**) GSEA enrichment plots of immune-related phenotype in low TMB patients compared with that in high TMB patients. (**C**) Correlation analyses between survival time and risk score based on TMB level. (**D**) Risk score distribution in high- and low-TMB groups. Data are presented as mean ± SEM. (**E**) Kaplan–Meier curves showing favorable survival in patients with high TMB. (**F**) Kaplan–Meier curves showing significantly prolonged survival in patients with high TMB and low risk compared with patients with low TMB and high risk.

To date, higher TMB has been validated prospectively and emerged as a critical biomarker for patient selection in NSCLC^12^. Previously, we reported a similar founding that TMB was significantly correlated with immunotherapeutic outcome^16^. To further illuminate the mechanism underlying immune infiltration and survival in LUSC, we associated TMB and the aforesaid immune-related gene signature. We visualized the somatic mutation data utilizing the maftools R package. Here we depicted a waterfall distribution of mutation information of genes in patients from the TCGA dataset, where annotations with various colors indicate the different patterns of the mutation (Fig. 4A). We calculated the count of nonsynonymous mutations per Mb and further divided patients into two groups with high- and low-TMB levels using 4.1 mutations/Mb as the cutoff value. GSEA analysis revealed that low TMB patients were prominent enriched in immune-related biological processes: Leukocyte migration (NES = 1.87, p = 0.002), Cellular defense response (NES = 1.86, p = 0.032), Inflammatory response (NES = 1.80, p = 0.014), Immune response (NES = 1.76, p = 0.045) (Fig. 4B) (Table S6). Conversely, none of the immune-related biological processes were enriched in high TMB patients (Table S7).

Additionally, correlation analyses exhibited that risk score was pronouncedly negatively correlated with overall survival in patients with high TMB and low TMB (Fig. 4C). Consistently, a significant difference was observed between the two groups (Fig. 4D). Similar to the IPM, we found that patients with low levels of TMB had poor survival (Fig. 4E). Combining the risk score of IPM with TMB level, patients with low levels of TMB and high immune risk suffered significantly shorter survival compared to those with high TMB and low immune risk (Fig. 4F).

### The IPM is an independent prognostic factor

**Figure 5:**
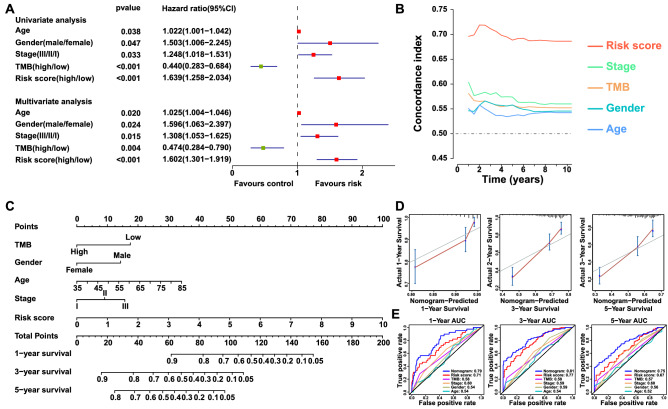
Nomogram development and validation based on the IPM. (**A**) Univariate and multivariate Cox regression analysis of the prognostic value of clinical features and the IPM in TCGA LUSC cohort. Red squares indicate adverse outcomes, and green squares indicate favorable outcomes. (**B**) C-index showing the prognostic performance of clinical features and the IPM. (**C**) Nomogram integrating clinical characteristics with the IPM for predicting 1-,3-, 5-year survival probability. (**D**) Calibration plot for internal validation of the nomogram. (**E**) Time-dependent ROC curves for evaluating the predictive efficacy of the nomogram.

Univariate and multivariate Cox regression analyses were performed to determine whether the IPM acts as an independent prognostic indicator for patients with LUSC. Clinical factors including TMB, age, gender, stage, and IPM were closely related to patient survival (Fig. 5A). After adjusting for other clinical parameters, the IPM remained as an independent significant predictor for LUSC prognosis (HR 1.602, 95% CI 1.301–1.919, p < 0.001) (Fig. 5A). Besides, the expression levels of IRGs were significantly associated with age, gender, and stage (Fig. S6). Furthermore, C-index values were calculated to compare the prognostic performance of each characteristic. C-index estimates the prediction concordance between predicted and actual survival, which ranges from 0.5 (random estimation) to 1 (perfect discrimination)^39^. The IPM showed a higher mean C-index (0.670) than other clinical characteristics (0.524 to 0.549) (Fig. 5B). In summary, these data confirmed that the IPM possessed valuable predictive capacity independent of other clinical factors.

### Nomogram development and validation based on the IPM

Next, we established a nomogram that integrated clinical risk factors with the IPM to quantify the survival probability in LUSC patients (Fig. 5C). Consistent with our previous finding, the nomogram illustrated the IPM as the prevailing contribution to prognosis compared with conventional clinical characteristics. Each variable was allocated a score on the point scale. By adding together the total score, the probability of survival was available by drawing a line vertically down to the survival axis. With the same 1000 random splits, the C-index of the nomogram was 0.735 (95% CI 0.691–0.779), which showed good concordance between prediction and observation (Fig. 5D). In addition, the ROC curve was delineated to compare nomogram predictive efficacy with other prognostic factors. Likewise, the nomogram exhibited the largest AUC (Fig. 5E). The AUC of the nomogram for overall survival was 0.79 at 1-year, 0.81 at 3-years, 0.75 at 5-years, respectively. In conclusion, the nomogram displayed better predictive power in both short- and long-term survival in LUSC patients relative to individual prognostic factors.

## Discussion

Patients with early-stage and local advanced LUSC are at considerable risk of relapse and death, even after complete surgical excision. With the great advances in immunotherapy, current cancer-related researches focus on the essential role of the immune system. Reliable prognostic biomarkers based on immune characteristics are demanded to predict the risk of cancer. Previous studies have proposed immune-related signatures for survival stratification in NSCLC^18,40–43^. However, the further discrimination of LUSC patients by local immune features remains poorly understood. To our knowledge, this study firstly systematic reports the attributes and clinical significance of the IPM in patients with stage I–III LUSC.

In this study, we established a prognostic model based on twenty-four IRGs and validated it in multiple public datasets to describe the immune status of LUSC patients and explore their prognostic potential. The IPM stratified patients into groups with markedly different survival benefits. We could identify patients with LUSC who probably carried a high risk of poor prognosis. Further, integrated analysis combing the IPM with clinical factors was performed to provide a more precise evaluation of overall survival in LUSC.

The immune system can both restrain or facilitate malignant progression, which proceeds in three phases denominated as elimination, equilibrium, and escape according to the hypothesis of cancer immunoediting^44^. During the escape phase, tumor develops the capacity to circumvent immune recognition which may result from the immunosuppressive state within the TME^44,45^. At the clinical level, evading immune destruction involves in the elevation of immunosuppressive cells (e.g., TAMs and Treg cells), the expression of immune checkpoint molecules (e.g., CTLA-4 and PD-L1), and the reduction of mutation-derived tumor neoantigens^46–48^. Release from immunosuppression will open the door for immunotherapy that directly stimulate the antitumor immune response. Here, we identified specific populations of immune cells between high- and low-risk patients as well as possible mechanisms to enhance the antitumoral activity. Generally, high-risk LUSC patients possessed a higher proportion of immune suppressive cells such as macrophages M0, while a lower proportion of immune effector cells such as T cells CD8 and plasma cells. These results also increased the reliability of the predictive value of the IPM.

Moreover, the expression of critical TAM targets (CD47, CD73, SIRPA, and TIM-3) was significantly upregulated in the high-risk group. As previously described, TAMs induce immune suppression through various approaches, including negatively modulating the activation of T cells and NK cells^49^, expressing immune checkpoint ligands that directly constrain T cell functions^50^, and releasing chemokines to recruit Treg cells in the TME^51^. Notably, CD47 expression on tumors shuts down macrophages by binding to SIRPA to avoid phagocytosis, termed do not eat me signal^52^. CD73, a newly admitted immune checkpoint mediator, is highly expressed on tumors and multiple cellular components in TME and inhibits the antitumor immune response^47^. The interaction between TIM-3 with immune cell signaling components facilitates the suppression of antitumor immunity^53^. Therefore, in this context, the risk score identified the immune checkpoints compatible with the power of tumor-infiltrating immune cells, indicating that the poor clinical outcome of patients with high risk may as a consequence of the immunosuppressive environment. Based on the above findings, immune dysregulation may be responsible for the differential prognoses between patient groups identified by our prognostic model.

A minority of tumor-specific mutations can lead to the formation of neoantigens that are recognized and targeted by the immune system, and TMB can act as an effective estimation of tumor neoantigen load^54^. In NSCLC patients receving immunotherapy, high TMB presents a strong correlation with improved response and durable benefit^14^. In this study, we demonstrated that the high-risk group showed lower TMB, which correlate with worse survival. When further stratified according to the TMB levels and risk score of IPM, patients with high TMB and low risk had a remarkable improved overall survival, underlying the intense immune response triggered by somatic mutations.

By integrating clinical risk factors with the IPM, we established a nomogram that displayed better survival predictive power and potential utility in guiding clinical use. For patients with early-stage LUSC, neoadjuvant and adjuvant immune checkpoint inhibitor therapies showed promising results in survival benefit^55^. Revealing the status of patients’ immune response using standardized immune assays is becoming an essential requirement to guide the optimal therapeutic intervention^56^. The development of novel immunotherapy strategies should focus on the tumor-infiltrating immune cell network instead of targeting a single kind of immune cell or immune-related gene. The IPM-based nomogram may have the potential to estimate prognosis and provide insight into guiding individualized clinical management for LUSC patients after surgery.

Some limitations of our study need to be acknowledged include its retrospective nature, and transcriptomic analysis cannot provide a complete molecular picture of the immune environment. In addition, further biological experiments are required for the rigorous validation of the IPM in LUSC.

In summary, the proposed IPM is a promising biomarker for the prediction and stratification of stage I–III LUSC patients. It was proven to act as an independent prognostic factor and reflect the overall intensity of the immune response. This work demonstrates an immune-related model associated with stage I–III LUSC and may contribute to the individualized management. Further, prospective studies are warranted to verify the predictive efficacy of IPM for LUSC.

## Supplementary Information

Supplementary Information.
