# Supplementary material for: Development and validation of an individualized immune prognostic model in stage I–III lung squamous cell carcinoma
Source: Sci Rep. 2021 Jun 16;11:12727. doi: 10.1038/s41598-021-92115-0 (PMC8209222; doi:10.1038/s41598-021-92115-0)
Supplement: Supplementary file 1 — Supplementary Information. [file 41598_2021_92115_MOESM1_ESM.docx]

SUPPLEMENTARY MATERIAL

Development and validation of an individualized immune prognostic model in stage I–III lung squamous cell carcinoma

Qi-Fan Yang^1^ M.D.^#^, Di Wu^1^ M.D.^#^, Jian Wang^1^ M.D., Li Ba^2^ M.D., Chen Tian^1^ M.D., Yu-Ting Liu^1^ M.D., Yue Hu^1,^* M.D., Li Liu^1,^* M.D., Ph.D.

^1^ Cancer Center, Union Hospital, Tongji Medical College, Huazhong University of Science and Technology, Wuhan 430022, China

^2^ Department of Ultrasound, Union Hospital, Tongji Medical College, Huazhong University of Science and Technology, Wuhan 430022, China

^#^ These authors contributed equally: Qi-Fan Yang and Di Wu.

*Correspondence:

Li Liu, M.D., Ph.D.

Cancer Center, Union Hospital, Tongji Medical College, Huazhong University of Science and Technology, Wuhan 430022, China

E-mail: [liulist2013@163.com](mailto:liulist2013@163.com)

Yue Hu, M.D.

Cancer Center, Union Hospital, Tongji Medical College, Huazhong University of Science and Technology, Wuhan 430022, China

E-mail: [huyue_cmu@126.com](mailto:huyue_cmu@126.com)

Supplementary Figures


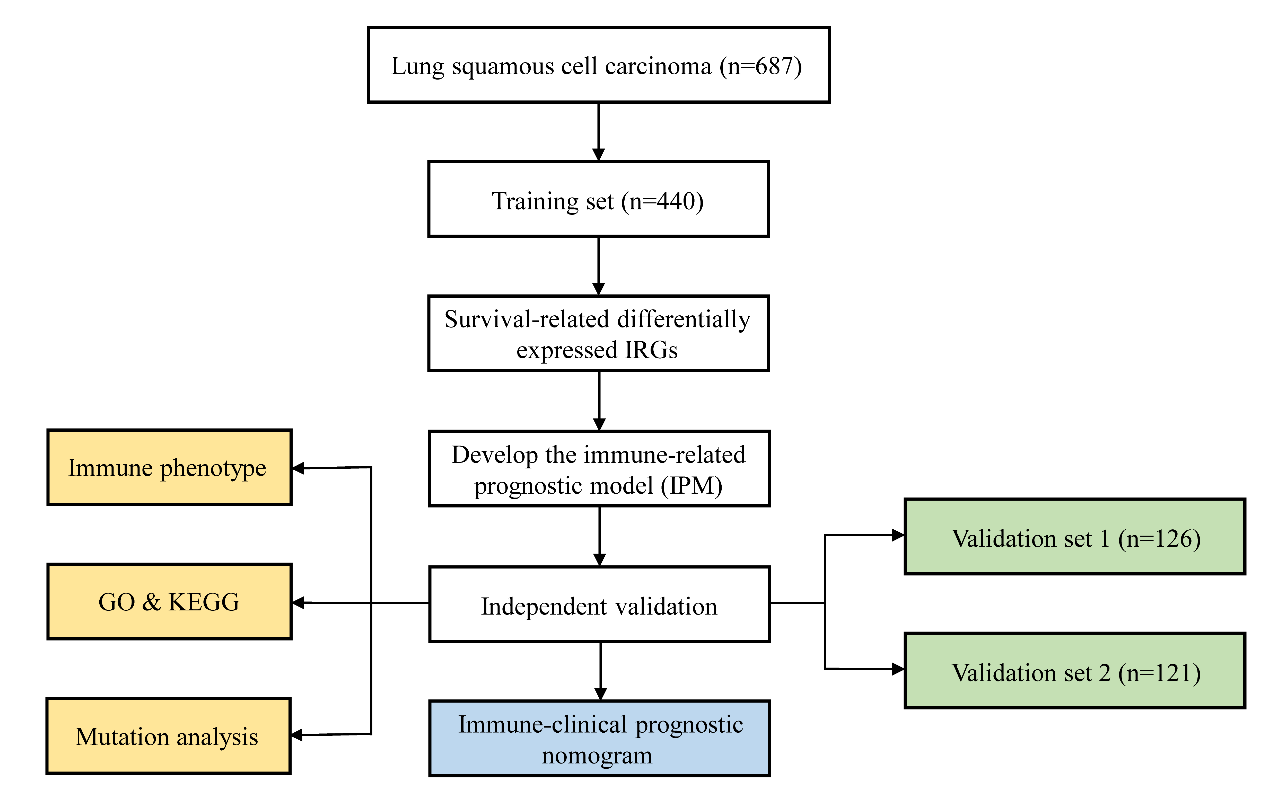


Figure S1 Overview of study design.


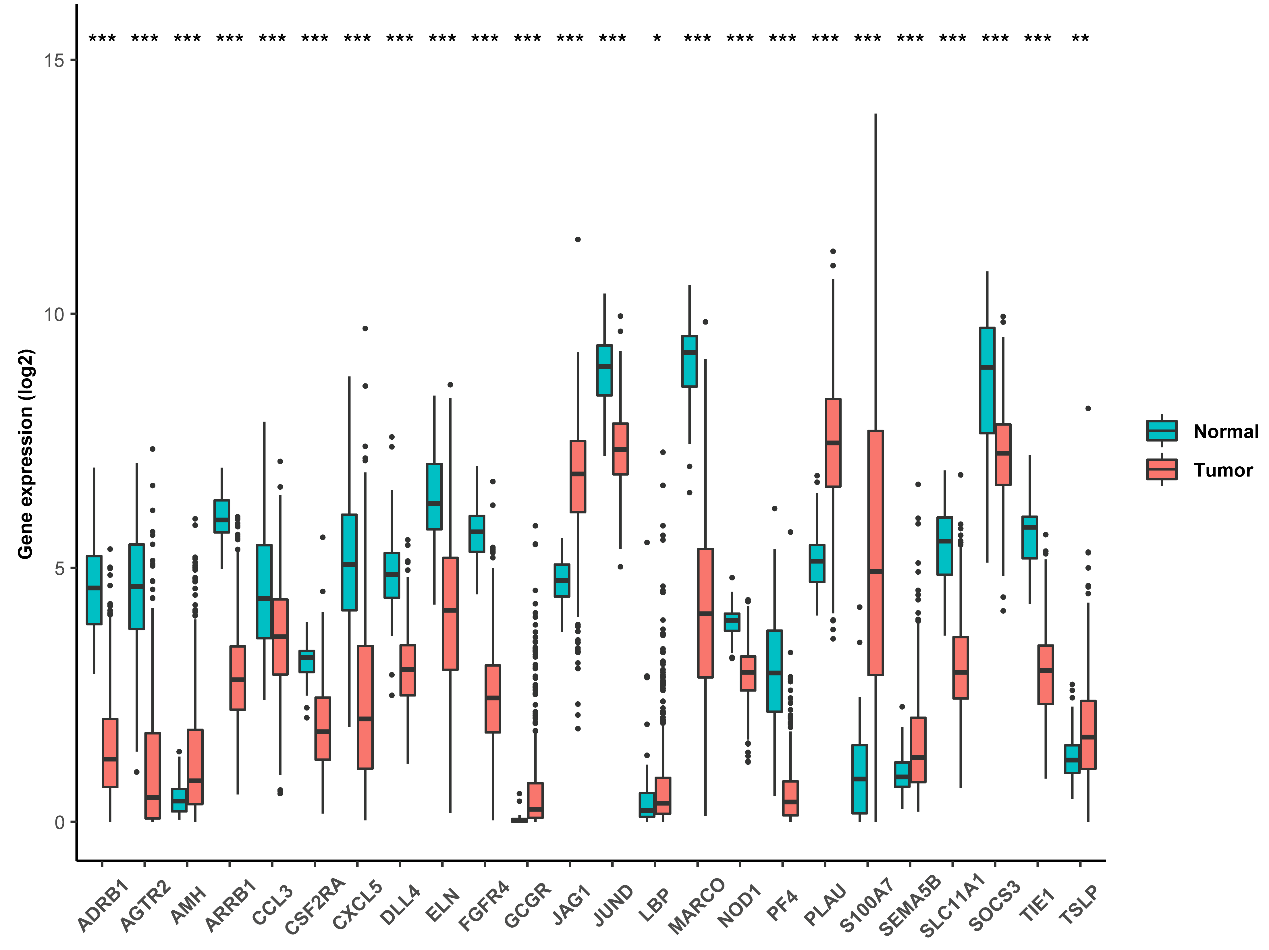


Figure S2 Expression of IRGs. Twenty-four IRGs used to establish the IPM were differentially expressed in LUSC compared to normal samples. (*, p value < 0.05; **, p value < 0.01; ***, p value < 0.001).


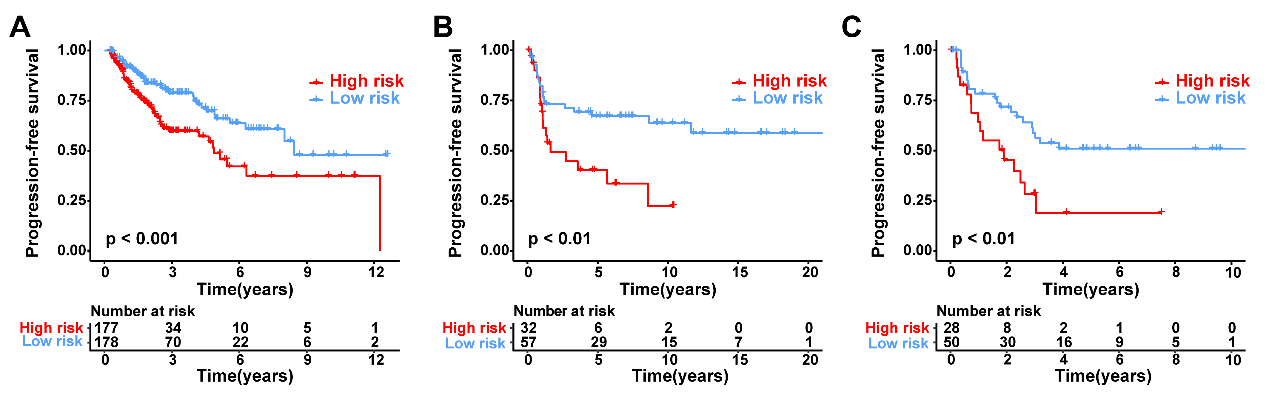


Figure S3. Progression-free survival of LUSC patients with high risk and low risk. Kaplan-Meier plots according to the IPM in the training set (A), validation set 1 (B) and validation set 2 (C).


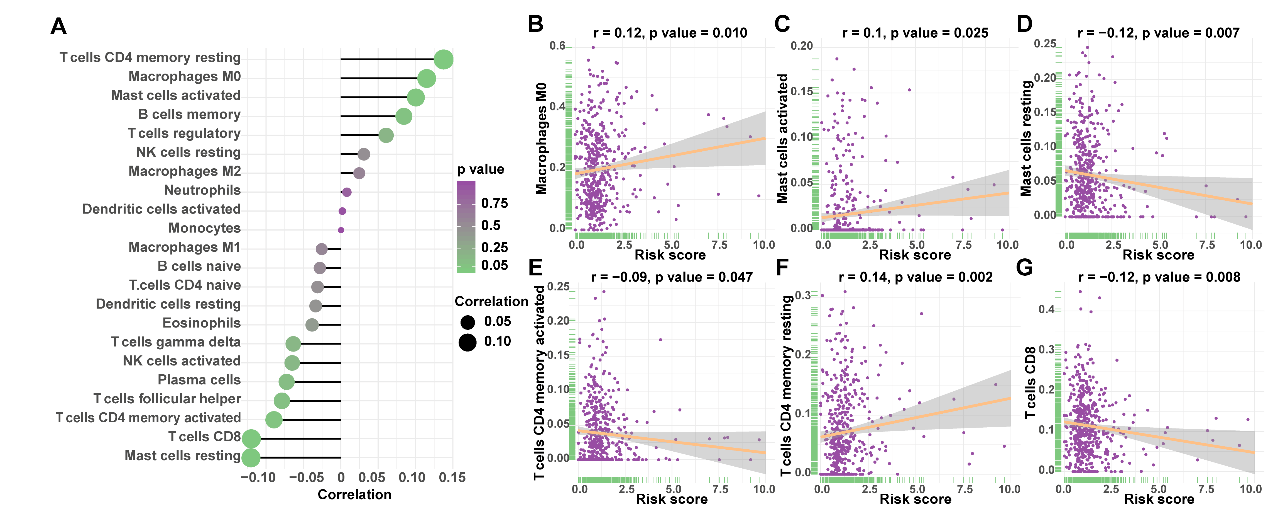


Figure S4 Correlation between risk score and immune cells. (A) Correlation between risk score and 22 infiltrating immune cell subtypes. (B–G) Scatter plot of risk score and significantly relevant immune cell subtypes.


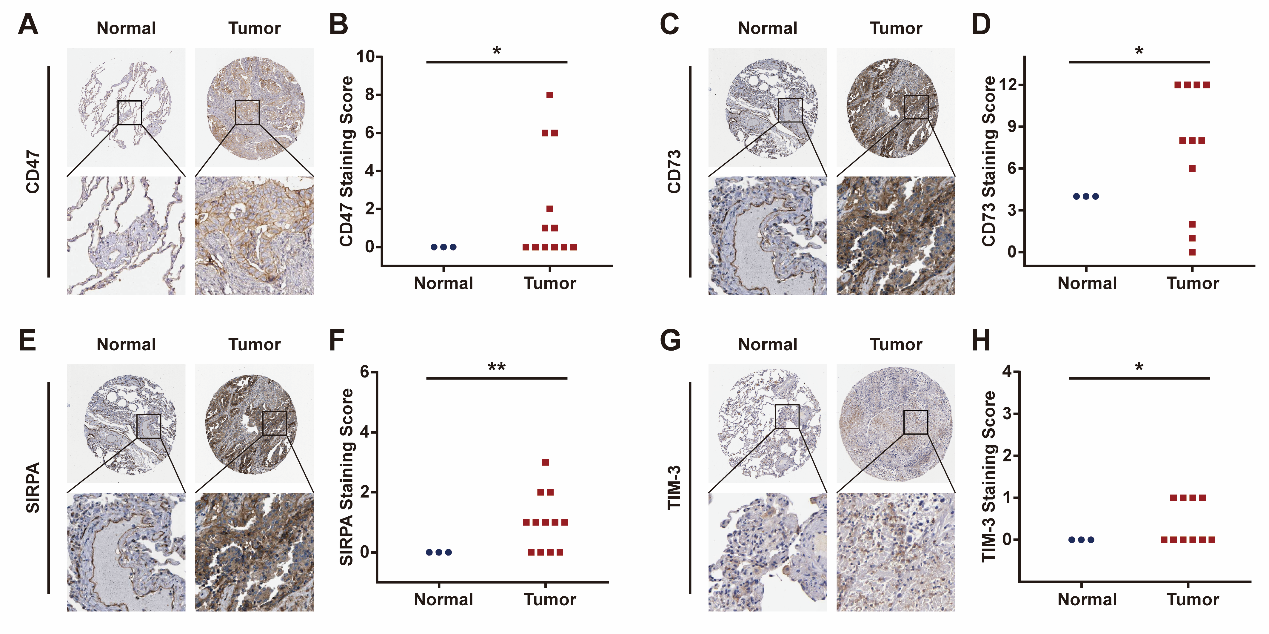


Figure S5 Immunohistochemistry validation of TAM target. Immunohistochemistry staining results validated from the Human Protein Atlas database revealed the CD47 (A-B), CD73 (C-D), SIRPA (E-F) and TIM-3 (G-H) protein to be upregulated in lung cancer tissues compared to normal lung tissues. (*, p value < 0.05; **, p value < 0.01; ***, p value < 0.001).


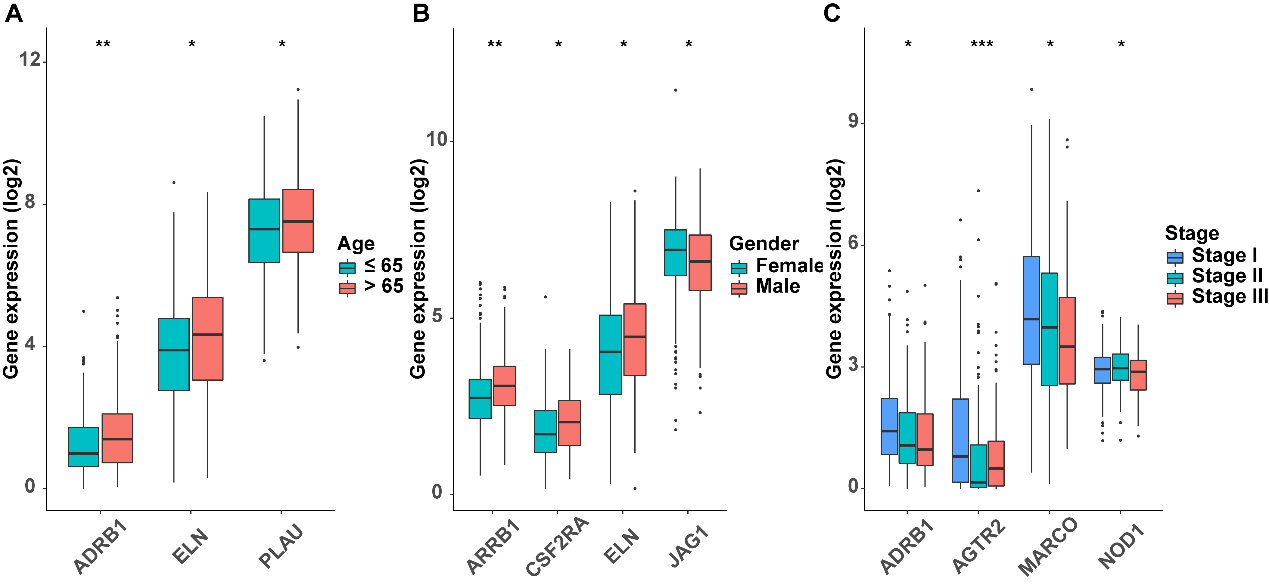


Figure S6 Clinical significance of IRGs. The expression levels of IRGs were significantly associated with age, gender and stage. (*, p value < 0.05; **, p value < 0.01; ***, p value < 0.001).

Supplementary Tables

Table S1 Characteristics of study populations in public datasets.

|  | Training set |  | Validation set 1 | |  | Validation set 2 | | *p* Value^*^ | |  |
| --- | --- | --- | --- | --- | --- | --- | --- | --- | --- | --- |
| Source Database | TCGA |  | GSE30219 | GSE37745 |  | GSE41271 | GSE42127 | |  | |
| Platform | Illumina HiSeq |  | Affymetrix HGU133+2.0 | Affymetrix HGU133+2.0 |  | Illumina HumanWG-6 v3.0 | Illumina HumanWG-6 v3.0 | |  | |
| Total | 440 |  | 60 | 66 |  | 78 | 43 | |  | |
| Age |  |  |  |  |  |  |  | | .063 | |
| Mean (SD) | 67.2(8.6) |  | 63.5(8.7) | 66.1(8.7) |  | 66.2(8.0) | 68.1(7.7) | |  | |
| Range | 39-85 |  | 46-82 | 40-84 |  | 46-84 | 52-84 | |  | |
| Gender |  |  |  |  |  |  |  | | .001 | |
| Male | 328(74.5) |  | 55(91.7) | 46(69.7) |  | 48(61.5) | 25(58.1) | |  | |
| Female | 112(25.5) |  | 5(8.3) | 20(30.3) |  | 30(38.5) | 18(41.9) | |  | |
| TNM stage |  |  |  |  |  |  |  | | .000 | |
| Stage I | 220(50.0) |  | 49(81.7) | 40(60.6) |  | 30(38.5) | 23(53.5) | |  | |
| Stage II | 149(33.9) |  | 7(11.7) | 15(22.7) |  | 17(21.8) | 10(23.3) | |  | |
| Stage III | 71(16.1) |  | 4(6.7) | 11(16.7) |  | 31(39.7) | 10(23.3) | |  | |
| Histology |  |  |  |  |  |  |  | |  | |
| Squamous carcinoma | 425(100.0) |  | 60(100.0) | 66(100.0) |  | 78(100.0) | 43(100.0) | |  | |
| Median Follow-up in years | 3.3 |  | 16.6 | 8.7 |  | 5.3 | 6.2 | | .947 | |
| No. of death | 187(42.5) |  | 42(70) | 52(78.8) |  | 41(52.6) | 21(48.8) | |  | |

Abbreviations: TCGA, TCGA lung squamous cell carcinoma dataset;

^*^The difference between training and validation sets was calculated in terms of clinical pathologic factors. Specifically, age
 was compared with Kruskal Wallis test; gender and stage was compared with Pearsonχ2 test; follow-up difference was
 assessed with log-rank test.

Table S2 Univariable Cox regression analyses of prognostic differentially expressed IRGs in the training set.

| Gene | HR(95%CI) | p value | ID | Name | Category |
| --- | --- | --- | --- | --- | --- |
| HLA-DRB5 | 1.1326(1.0038-1.2780) | 0.043233339 | 3127 | major histocompatibility complex, class II, DR beta 5 | Antigen_Processing_and_Presentation |
| SHFM1 | 0.8193(0.6822-0.9839) | 0.032912086 | 7979 | split hand/foot malformation type 1 | Antigen_Processing_and_Presentation |
| SFTPD | 1.1343(1.0051-1.2802) | 0.041192739 | 6441 | surfactant protein D | Antimicrobials |
| S100A7 | 1.1527(1.0199-1.3028) | 0.022889017 | 6278 | S100 calcium binding protein A7 | Antimicrobials |
| S100A5 | 0.8081(0.6600-0.9894) | 0.039069154 | 6276 | S100 calcium binding protein A5 | Antimicrobials |
| MMP9 | 1.1117(1.0048-1.2299) | 0.040101884 | 4318 | matrix metallopeptidase 9 | Antimicrobials |
| LBP | 1.0964(1.0086-1.1918) | 0.030642809 | 3929 | lipopolysaccharide binding protein | Antimicrobials |
| SFTPA1 | 1.1389(1.0070-1.2880) | 0.038340522 | 653509 | surfactant protein A1 | Antimicrobials |
| TLR3 | 1.1626(1.0158-1.3306) | 0.028741122 | 7098 | toll-like receptor 3 | Antimicrobials |
| SOCS3 | 1.1439(1.0089-1.2969) | 0.035851729 | 9021 | suppressor of cytokine signaling 3 | Antimicrobials |
| ELN | 1.1319(1.0074-1.2719) | 0.037220347 | 2006 | elastin | Antimicrobials |
| NOD1 | 1.2119(1.0549-1.3922) | 0.006614626 | 10392 | nucleotide-binding oligomerization domain containing 1 | Antimicrobials |
| TK2 | 1.2020(1.0391-1.3903) | 0.013257149 | 7084 | thymidine kinase 2, mitochondrial | Antimicrobials |
| DLL4 | 1.1904(1.0417-1.3603) | 0.010467059 | 54567 | delta-like 4 | Antimicrobials |
| SLC11A1 | 1.1296(1.0055-1.2690) | 0.040177397 | 6556 | solute carrier family 11, member 1 | Antimicrobials |
| MARCO | 1.1528(1.0314-1.2884) | 0.012241845 | 8685 | macrophage receptor with collagenous structure | Antimicrobials |
| JUND | 1.1834(1.0540-1.3286) | 0.004371946 | 3727 | jun D proto-oncogene | Antimicrobials |
| RNASE7 | 1.2153(1.0940-1.3500) | 0.000277826 | 84659 | ribonuclease, RNase A family, 7 | Antimicrobials |
| ARRB1 | 1.1319(1.0029-1.2776) | 0.044811547 | 408 | arrestin, beta 1 | Antimicrobials |
| ANXA6 | 1.1566(1.0147-1.3185) | 0.029403296 | 309 | annexin A6 | Antimicrobials |
| AGER | 1.1185(1.0098-1.2390) | 0.031739663 | 177 | advanced glycosylation end product-specific receptor | Antimicrobials |
| JUN | 1.1827(1.0520-1.3297) | 0.004976724 | 3725 | jun oncogene | BCRSignalingPathway |
| CD81 | 1.1666(1.0153-1.3404) | 0.029649955 | 975 | CD81 molecule | BCRSignalingPathway |
| FOS | 1.1796(1.0580-1.3151) | 0.0029187 | 2353 | v-fos FBJ murine osteosarcoma viral oncogene homolog | BCRSignalingPathway |
| LILRB3 | 1.1412(1.0053-1.2954) | 0.04122458 | 11025 | leukocyte immunoglobulin-like receptor, subfamily B , member 3 | BCRSignalingPathway |
| EDNRB | 1.1551(1.0486-1.2723) | 0.00347279 | 1910 | endothelin receptor type B | Chemokine_Receptors |
| PLXND1 | 1.1905(1.0360-1.3680) | 0.013971473 | 23129 | plexin D1 | Chemokine_Receptors |
| PPBP | 1.1421(1.0348-1.2606) | 0.008328053 | 5473 | pro-platelet basic protein | Chemokines |
| CXCL5 | 1.1558(1.0508-1.2713) | 0.002885278 | 6374 | chemokine (C-X-C motif) ligand 5 | Chemokines |
| PF4 | 1.1685(1.0588-1.2895) | 0.00195846 | 5196 | platelet factor 4 | Chemokines |
| CCL13 | 1.1825(1.0226-1.3674) | 0.023750895 | 6357 | chemokine (C-C motif) ligand 13 | Chemokines |
| PLAU | 1.3620(1.1932-1.5547) | 4.75473E-06 | 5328 | plasminogen activator, urokinase | Chemokines |
| CCL3 | 1.2083(1.0466-1.3950) | 0.009834173 | 6348 | chemokine (C-C motif) ligand 3 | Chemokines |
| CYR61 | 1.1339(1.0083-1.2753) | 0.035988511 | 3491 | cysteine-rich, angiogenic inducer, 61 | Chemokines |
| SEMA4C | 1.1736(1.0618-1.2972) | 0.001725108 | 54910 | sema domain, immunoglobulin domain (Ig), transmembrane domain (TM) and short cytoplasmic domain, (semaphorin) 4C | Chemokines |
| SEMA5B | 1.1432(1.0181-1.2836) | 0.023565922 | 54437 | sema domain, seven thrombospondin repeats (type 1 and type 1-like), transmembrane domain (TM) and short cytoplasmic domain, (semaphorin) 5B | Chemokines |
| ACVRL1 | 1.1830(1.0392-1.3465) | 0.01100785 | 94 | activin A receptor type II-like 1 | Cytokine_Receptors |
| ADRB1 | 1.1823(1.0383-1.3463) | 0.011507018 | 153 | adrenergic, beta-1-, receptor | Cytokine_Receptors |
| AGTR2 | 1.1470(1.0488-1.2544) | 0.002663787 | 186 | angiotensin II receptor, type 2 | Cytokine_Receptors |
| CSF2RA | 1.1405(1.0147-1.2819) | 0.027477016 | 1438 | colony stimulating factor 2 receptor, alpha, low-affinity (granulocyte-macrophage) | Cytokine_Receptors |
| EPOR | 1.2140(1.0537-1.3988) | 0.007287757 | 2057 | erythropoietin receptor | Cytokine_Receptors |
| FGFR4 | 1.1623(1.0262-1.3164) | 0.017916036 | 2264 | fibroblast growth factor receptor 4 | Cytokine_Receptors |
| FLT4 | 1.2055(1.0472-1.3877) | 0.00924913 | 2324 | fms-related tyrosine kinase 4 | Cytokine_Receptors |
| GCGR | 0.5881(0.3792-0.9121) | 0.017743799 | 2642 | glucagon receptor | Cytokine_Receptors |
| NR4A1 | 1.1820(1.0447-1.3373) | 0.007962589 | 3164 | nuclear receptor subfamily 4, group A, member 1 | Cytokine_Receptors |
| NR4A3 | 1.1702(1.0539-1.2994) | 0.003251866 | 8013 | nuclear receptor subfamily 4, group A, member 3 | Cytokine_Receptors |
| SDC4 | 1.1324(1.0034-1.2781) | 0.043901107 | 6385 | syndecan 4 | Cytokine_Receptors |
| TIE1 | 1.1372(1.0039-1.2882) | 0.043202993 | 7075 | tyrosine kinase with immunoglobulin-like and EGF-like domains 1 | Cytokine_Receptors |
| TNFSF11 | 1.1533(1.0157-1.3095) | 0.027781807 | 8600 | tumor necrosis factor (ligand) superfamily, member 11 | Cytokines |
| AMH | 0.7636(0.6112-0.9539) | 0.01754262 | 268 | anti-Mullerian hormone | Cytokines |
| APLN | 1.1770(1.0384-1.3339) | 0.010756396 | 8862 | apelin | Cytokines |
| BMP2 | 1.1279(1.0019-1.2697) | 0.046366732 | 650 | bone morphogenetic protein 2 | Cytokines |
| CSF2 | 1.1415(1.0445-1.2474) | 0.003491459 | 1437 | colony stimulating factor 2 (granulocyte-macrophage) | Cytokines |
| JAG1 | 1.1778(1.0541-1.3161) | 0.003858836 | 182 | jagged 1 (Alagille syndrome) | Cytokines |
| LIF | 1.1437(1.0093-1.2960) | 0.035220957 | 3976 | leukemia inhibitory factor (cholinergic differentiation factor) | Cytokines |
| OGN | 1.1562(1.0137-1.3187) | 0.030608975 | 4969 | osteoglycin | Cytokines |
| PDGFB | 1.1946(1.0576-1.3493) | 0.004221913 | 5155 | platelet-derived growth factor beta polypeptide (simian sarcoma viral (v-sis) oncogene homolog) | Cytokines |
| PNOC | 1.1226(1.0202-1.2353) | 0.017785134 | 5368 | prepronociceptin | Cytokines |
| RETN | 1.1008(1.0149-1.1940) | 0.020486179 | 56729 | resistin | Cytokines |
| TNFSF14 | 1.1358(1.0127-1.2739) | 0.029585332 | 8740 | tumor necrosis factor (ligand) superfamily, member 14 | Cytokines |
| TSLP | 0.4151(0.2194-0.7856) | 0.006904242 | 85480 | thymic stromal lymphopoietin | Cytokines |
|  |  |  |  |  |  |

Table S3 Coefficients and multivariable Cox model results of IRGs.

| Gene | Coefficients | HR(95%CI) | p value |
| --- | --- | --- | --- |
| CXCL5 | 0.1626 | 1.1766(1.0471-1.3221) | 0.0063 |
| PF4 | 0.1216 | 1.1293(0.9998-1.2756) | 0.0503 |
| S100A7 | 0.1707 | 1.1861(1.0425-1.3495) | 0.0095 |
| PLAU | 0.3121 | 1.3663(1.1708-1.5945) | 0.0001 |
| LBP | 0.0974 | 1.1023(1.0028-1.2118) | 0.0436 |
| SOCS3 | -0.1357 | 0.8731(0.7262-1.0496) | 0.1485 |
| ELN | 0.1447 | 1.1557(1.0036-1.3307) | 0.0444 |
| NOD1 | 0.1719 | 1.1875(0.9937-1.4191) | 0.0586 |
| DLL4 | 0.3691 | 1.4465(1.0913-1.9174) | 0.0102 |
| SLC11A1 | -0.4409 | 0.6435(0.4936-0.8389) | 0.0011 |
| MARCO | 0.3177 | 1.3740(1.0786-1.7503) | 0.0101 |
| JUND | 0.2256 | 1.2531(1.0755-1.4600) | 0.0038 |
| ARRB1 | -0.2572 | 0.7732(0.6063-0.9861) | 0.0382 |
| CCL3 | 0.1453 | 1.1564(0.9569-1.3975) | 0.1326 |
| SEMA5B | 0.1729 | 1.1887(1.0537-1.3410) | 0.0049 |
| AMH | -0.1932 | 0.8243(0.6497-1.0459) | 0.1118 |
| JAG1 | 0.1691 | 1.1843(1.0688-1.3122) | 0.0012 |
| TSLP | -0.8942 | 0.4089(0.2025-0.8258) | 0.0126 |
| ADRB1 | 0.1740 | 1.1901(1.0212-1.3870) | 0.0259 |
| AGTR2 | 0.2185 | 1.2442(1.0811-1.4319) | 0.0023 |
| CSF2RA | 0.2550 | 1.2905(1.0657-1.5627) | 0.0090 |
| FGFR4 | 0.1968 | 1.2175(1.0670-1.3893) | 0.0035 |
| GCGR | -0.6143 | 0.5410(0.3242-0.9029) | 0.0187 |
| TIE1 | -0.4342 | 0.6478(0.4764-0.8808) | 0.0056 |
|  |  |  |  |

Table S4 Analysis of correlations between risk score and immune checkpoints.

| Variable 1 | Variable 2 | Pearson correlation coefficient | p value |
| --- | --- | --- | --- |
| Risk score | TIM3 | 0.1138 | 0.0169 |
| Risk score | CD47 | 0.1585 | 0.0008 |
| Risk score | SIRPA | 0.1624 | 0.0006 |
| Risk score | CD73 | 0.3145 | <0.0001 |
| TIM3 | CD47 | 0.242 | <0.0001 |
| TIM3 | SIRPA | 0.4002 | <0.0001 |
| TIM3 | CD73 | 0.1679 | 0.0004 |
| CD47 | SIRPA | 0.1747 | 0.0002 |
| CD47 | CD73 | 0.1161 | 0.0148 |
| SIRPA | CD73 | 0.1186 | 0.0128 |
|  |  |  |  |

Table S5 Function and pathway enrichment analysis of the twenty-four IRGs.

|  | ID | Gene function | Gene count | Gene | p value | FDR |
| --- | --- | --- | --- | --- | --- | --- |
| GO | GO:0032496 | response to lipopolysaccharide | 7 | CXCL5/PF4/S100A7/LBP/SLC11A1/JUND/CCL3 | 1.27E-07 | 4.54E-05 |
| GO | GO:0070372 | regulation of ERK1 and ERK2 cascade | 7 | S100A7/NOD1/MARCO/ARRB1/CCL3/FGFR4/TIE1 | 1.27E-07 | 4.54E-05 |
| GO | GO:0002237 | response to molecule of bacterial origin | 7 | CXCL5/PF4/S100A7/LBP/SLC11A1/JUND/CCL3 | 1.66E-07 | 4.54E-05 |
| GO | GO:0071621 | granulocyte chemotaxis | 5 | CXCL5/PF4/S100A7/LBP/CCL3 | 4.07E-07 | 8.56E-05 |
| GO | GO:0097530 | granulocyte migration | 5 | CXCL5/PF4/S100A7/LBP/CCL3 | 7.05E-07 | 1.20E-04 |
| GO | GO:0001819 | positive regulation of cytokine production | 7 | PF4/LBP/NOD1/SLC11A1/CCL3/85480/186 | 7.59E-07 | 1.20E-04 |
| GO | GO:0032760 | positive regulation of tumor necrosis factor production | 4 | PF4/LBP/NOD1/CCL3 | 2.24E-06 | 3.01E-04 |
| GO | GO:1903557 | neutrophil chemotaxis | 4 | CXCL5/PF4/LBP/CCL3 | 2.63E-06 | 3.01E-04 |
| GO | GO:0097529 | myeloid leukocyte migration | 5 | CXCL5/PF4/S100A7/LBP/CCL3 | 4.94E-06 | 4.79E-04 |
| GO | GO:0030595 | leukocyte chemotaxis | 5 | CXCL5/PF4/S100A7/LBP/CCL3 | 7.53E-06 | 6.78E-04 |
| KEGG | hsa04060 | Cytokine-cytokine receptor interaction | 6 | CXCL5;PF4;CCL3;AMH;TSLP;CSF2RA | 2.34E-04 | 0.076300876 |
| KEGG | hsa04062 | Chemokine signaling pathway | 4 | CXCL5;PF4;ARRB1;CCL3 | 2.66E-03 | 0.323880365 |
| KEGG | hsa04657 | IL-17 signaling pathway | 3 | CXCL5;S100A7;JUND | 2.98E-03 | 0.323880365 |
| KEGG | hsa04668 | TNF signaling pathway | 3 | CXCL5;SOCS3;JAG1 | 4.79E-03 | 0.390098033 |
| KEGG | hsa04330 | Notch signaling pathway | 2 | DLL4;JAG1 | 9.88E-03 | 0.644447117 |
| KEGG | hsa04630 | JAK-STAT signaling pathway | 3 | SOCS3;TSLP;CSF2RA | 1.39E-02 | 0.753121459 |
| KEGG | hsa05133 | Pertussis | 2 | CXCL5;NOD1 | 2.37E-02 | 1 |
| KEGG | hsa05132 | Salmonella infection | 2 | LBP;CCL3 | 2.98E-02 | 1 |
| KEGG | hsa05323 | Rheumatoid arthritis | 2 | CXCL5;CCL3 | 3.24E-02 | 1 |
| KEGG | hsa04658 | Th1 and Th2 cell differentiation | 2 | DLL4;JAG1 | 3.37E-02 | 1 |
|  |  |  |  |  |  |  |

Table S6 Top 20 gene enrichment in low TMB LUSCs.

| Name | Size | ES | NES | NOM  p-val | FDR  q-val | FWER p-val | Rank at max | Leading Edge |
| --- | --- | --- | --- | --- | --- | --- | --- | --- |
| LIPID_RAFT | 27 | 0.66364 | 2.0274 | 0 | 0.164 | 0.118 | 2790 | tags=52%, list=19%, signal=64% |
| REGULATION_OF_HEART_CONTRACTION | 16 | 0.69296 | 2.00393 | 0.0025 | 0.118 | 0.144 | 2518 | tags=56%, list=17%, signal=67% |
| HALLMARK_KRAS_SIGNALING_UP | 188 | 0.47554 | 1.90133 | 0.0028 | 0.255 | 0.318 | 3083 | tags=49%, list=21%, signal=61% |
| PROTEASE_INHIBITOR_ACTIVITY | 29 | 0.61007 | 1.86895 | 0.0104 | 0.266 | 0.379 | 2211 | tags=41%, list=15%, signal=48% |
| LEUKOCYTE_MIGRATION | 22 | 0.64936 | 1.86631 | 0.0025 | 0.217 | 0.382 | 1965 | tags=55%, list=13%, signal=63% |
| CELLULAR_DEFENSE_RESPONSE | 41 | 0.64032 | 1.86107 | 0.032 | 0.191 | 0.395 | 1459 | tags=46%, list=10%, signal=51% |
| DEFENSE_RESPONSE | 215 | 0.51616 | 1.84532 | 0.0239 | 0.191 | 0.434 | 2411 | tags=45%, list=16%, signal=52% |
| RESPONSE_TO_WOUNDING | 163 | 0.49173 | 1.8385 | 0.0102 | 0.179 | 0.45 | 2777 | tags=47%, list=18%, signal=57% |
| HALLMARK_COAGULATION | 116 | 0.50402 | 1.80192 | 0.0098 | 0.223 | 0.56 | 3236 | tags=52%, list=22%, signal=65% |
| INFLAMMATORY_RESPONSE | 111 | 0.50722 | 1.79878 | 0.0145 | 0.206 | 0.567 | 2777 | tags=47%, list=18%, signal=57% |
| DEFENSE_RESPONSE_TO_BACTERIUM | 16 | 0.6511 | 1.79639 | 0.0167 | 0.191 | 0.571 | 2855 | tags=69%, list=19%, signal=85% |
| IMMUNE_RESPONSE | 197 | 0.54509 | 1.76333 | 0.0451 | 0.232 | 0.659 | 2305 | tags=51%, list=15%, signal=59% |
| RESPONSE_TO_BACTERIUM | 15 | 0.64594 | 1.76144 | 0.0091 | 0.218 | 0.663 | 1970 | tags=67%, list=13%, signal=77% |
| CAMP_MEDIATED_SIGNALING | 39 | 0.47221 | 1.75924 | 0.0154 | 0.207 | 0.673 | 1728 | tags=38%, list=11%, signal=43% |
| INTERLEUKIN_RECEPTOR_ACTIVITY | 18 | 0.7527 | 1.74637 | 0.0242 | 0.216 | 0.701 | 2707 | tags=78%, list=18%, signal=95% |
| RESPONSE_TO_EXTERNAL_STIMULUS | 257 | 0.42974 | 1.73661 | 0.0158 | 0.218 | 0.721 | 2692 | tags=43%, list=18%, signal=52% |
| G_PROTEIN_SIGNALING_COUPLED_TO_CAMP_NUCLEOTIDE_SECOND_MESSENGER | 38 | 0.47424 | 1.73401 | 0.0207 | 0.209 | 0.722 | 1728 | tags=39%, list=11%, signal=44% |
| BLOOD_COAGULATION | 33 | 0.54498 | 1.73222 | 0.0227 | 0.2 | 0.724 | 2651 | tags=55%, list=18%, signal=66% |
| LOCOMOTORY_BEHAVIOR | 79 | 0.51577 | 1.72878 | 0.0337 | 0.194 | 0.726 | 2692 | tags=51%, list=18%, signal=61% |
| REGULATION_OF_SECRETION | 34 | 0.5168 | 1.72628 | 0.0182 | 0.188 | 0.733 | 2601 | tags=50%, list=17%, signal=60% |
|  |  |  |  |  |  |  |  |  |

Table S7. Top 20 gene enrichment in high TMB LUSCs.

| Name | Size | ES | NES | NOM  p-val | FDR  q-val | FWER p-val | Rank at max | Leading Edge |
| --- | --- | --- | --- | --- | --- | --- | --- | --- |
| M_PHASE | 104 | -0.70327 | -2.24022 | 0 | 0 | 0 | 1725 | tags=54%, list=11%, signal=60% |
| M_PHASE_OF_MITOTIC_CELL_CYCLE | 81 | -0.70722 | -2.20726 | 0 | 0.00131 | 0.001 | 1725 | tags=54%, list=11%, signal=61% |
| MITOSIS | 78 | -0.70527 | -2.20382 | 0 | 0.0012 | 0.002 | 2396 | tags=59%, list=16%, signal=70% |
| MITOTIC_CELL_CYCLE | 147 | -0.63999 | -2.19895 | 0 | 0.00142 | 0.003 | 1725 | tags=46%, list=11%, signal=51% |
| CELL_CYCLE_PHASE | 158 | -0.63436 | -2.16385 | 0 | 0.00282 | 0.005 | 1488 | tags=44%, list=10%, signal=49% |
| HALLMARK_G2M_CHECKPOINT | 189 | -0.70919 | -2.14395 | 0 | 0.00347 | 0.006 | 2665 | tags=68%, list=18%, signal=81% |
| CELL_CYCLE_PROCESS | 181 | -0.63382 | -2.14385 | 0 | 0.00298 | 0.006 | 1725 | tags=46%, list=11%, signal=51% |
| DNA_INTEGRITY_CHECKPOINT | 21 | -0.76427 | -2.11148 | 0 | 0.00494 | 0.014 | 1488 | tags=57%, list=10%, signal=63% |
| DNA_RECOMBINATION | 43 | -0.6792 | -2.108 | 0 | 0.00519 | 0.017 | 1350 | tags=53%, list=9%, signal=59% |
| CELL_CYCLE_GO_0007049 | 297 | -0.55065 | -2.08133 | 0 | 0.00646 | 0.023 | 1725 | tags=37%, list=11%, signal=41% |
| CELL_CYCLE_CHECKPOINT_GO_0000075 | 45 | -0.67898 | -2.0688 | 0 | 0.00704 | 0.03 | 1665 | tags=51%, list=11%, signal=57% |
| RNA_SPLICING | 89 | -0.68168 | -2.06555 | 0 | 0.00697 | 0.032 | 3251 | tags=74%, list=22%, signal=94% |
| REGULATION_OF_MITOTIC_CELL_CYCLE | 22 | -0.67988 | -2.06254 | 0 | 0.00681 | 0.032 | 1708 | tags=45%, list=11%, signal=51% |
| DOUBLE_STRANDED_DNA_BINDING | 29 | -0.71057 | -2.05414 | 0 | 0.00692 | 0.036 | 1500 | tags=48%, list=10%, signal=54% |
| RNA_PROCESSING | 167 | -0.62633 | -2.049 | 0 | 0.00691 | 0.038 | 3274 | tags=62%, list=22%, signal=78% |
| MEIOTIC_CELL_CYCLE | 29 | -0.72038 | -2.04795 | 0 | 0.00673 | 0.04 | 1292 | tags=52%, list=9%, signal=56% |
| HALLMARK_E2F_TARGETS | 195 | -0.72298 | -2.04586 | 0.002053 | 0.00648 | 0.041 | 2141 | tags=71%, list=14%, signal=81% |
| CHROMOSOME | 114 | -0.64611 | -2.04506 | 0 | 0.00618 | 0.042 | 1364 | tags=44%, list=9%, signal=48% |
| DOUBLE_STRAND_BREAK_REPAIR | 22 | -0.7372 | -2.0306 | 0 | 0.00719 | 0.054 | 1286 | tags=55%, list=9%, signal=60% |
| DNA_DAMAGE_RESPONSESIGNAL_TRANSDUCTION | 30 | -0.64587 | -2.02588 | 0 | 0.00716 | 0.057 | 2026 | tags=43%, list=13%, signal=50% |
|  |  |  |  |  |  |  |  |  |
